# 

**Published:** 1897-12-04

**Authors:** 


**Notices of Births, Deaths, and Marriages** are inserted in this column at a charge of 2s. 6d. for an announcement not exceeding four lines.

### NOTICE TO CORRESPONDENTS.

All MS., Letters, Books for Review, and other matters intended for the Editor should be addressed The Editor, "The Hospital," The Hospital Building, 28 & 29, Southampton Street, Strand, W.C. The Editor cannot undertake to return rejected MS., even when accompanied by stamped directed envelope.

**Notice to Advertisers and Subscribers.**

All Advertisements, Orders for Copies of The Hospital, and Business Communications should be addressed to **THE MANAGER, (Not to The Editor),** The Hospital, The Hospital Building, 28 & 29, Southampton Street, Strand, London, W.C.

The Cost of Subscription, post free, is as follows:—Per week, 2½d.; per quarter, 2s. 8d.; per half-year, 5s. 8d.; per annum, 10s. 6d. Foreign:—Per annum, 15s. 2d.; payable, in all cases, in advance.

**NOTICE.—Vol. XXII. of "The Hospital" (April, 1897, to September, 1897), in handsome cloth binding, is now on sale at the Office of this Journal, price 7s. 6d. Cases for binding the Numbers contained in this Volume, 1s. 6d. Index to Vol. XXII., 2½d., post free.**

**The Monthly Part for December is now ready, Price 6d., by post 8d.**

### BOOKS RECEIVED.

Macmillan and Co.
"Allbutt's System of Medicine." Vol. IV.
J. B. Lippincott and Co.
"Practice of Surgery." By Henry R. Wharton, M.D., and B. Farquhar Curtis, M.D.
A. D. Innes and Co.
"Drawing-room Plays for Children." By "Santos."
Sampson Low, Marston, and Co.
"Twentieth Century Practice." Vol. XI. Edited by Thomas L. Stedman, M.D.
W. H. and L. Collingridge.
"Guide to Printing and Publishing." Eleventh Edition.

**Periodicals and Pamphlets Received.**—Electrical Review, Girl's Own Paper, New Age, Industries and Iron, Woman's Signal, Scalpel, Literary Digest, Daylight.

### The Student of Medicine.

### Appointments.

N. H. Alcock, B.A., M.D., appointed Assistant to the Professor of Physiology, Trinity College, Dublin. A. W. Aldridge, M B., B.S.Durh., M.R.C.S.Eng., L.R.C.P.Lond., appointed Obstetric and Ophthalmic House Surgeon to the Queen's Hospital, Birmingham. Dr. W. Arnold, appointed Medical Officer for the Waltham District of the Melton Mowbray Union. E. Barclay-Smith, M.D., appointed Examiner in Human Anatomy to the University of Cambridge. W. M. Brown, M.B., C.M.Glasg., appointed Medical Officer for the Saltcoats District of the Stevenston Parish. Dr. Burland, appointed Medical Officer to the Burton Cottage Home of the Kettering Union. H. Crichton, M.B., B.S.Durh., appointed Senior House Surgeon to the Ingham Infirmary and South Shields and Westoe Dispensary. F. J. F. Culhane, M.R.C.S.Eng., L.S.A., appointed Medical Officer for the No. 4 (Great Marlow) and No. 12 (Little Marlow) Districts of the High Wycombe Union. A. Dingwall, M.B.Glasg., appointed Medical Officer of Health to the Presteigne Urban District Council. E. J. Dobbin, M.R.C.S.Eng., L.R.C.P.Lond., appointed Assistant Medical Officer of the Infirmary and the St. John's Road Workhouse of the Parish of St. Mary, Islington. W. Fulton, M.B., C.M.Glasg., appointed Medical Officer for the Stevenston District of the Stevenston Parish. H. Gervis, M.R.C.S., L.R.C.P., appointed House Surgeon to the Paddington Green Children's Hospital. C. J. Glasson, L.R.C.P.Lond., M.R.C.S Eng., appointed Medical Officer of Health for the Ellesmere Joint District. E. H. Houfton, M.B.Lond., M.R.C.S., appointed Medical Officer of Health to the Mansfield Woodhouse Urban District Council. A. A. Kanthack, M.D.Lond., F.R.C.S.Eng., appointed Professor of Pathology at Cambridge University. J. Kerr, M.A., M.D.Cantab., D.P.H., appointed Honorary Surgeon to the Bradford Eye and Ear Hospital. C. C. Lord, B.A.Cantab., M.R.C.S.Eng., L.R.C.P.Lond., appointed House Surgeon to the Queen's Hospital, Birmingham. Dr. A. Moore, appointed Medical Officer for the Rockcorry Dispensary District. W. A. Peverley, M.B., B.S.Durh , appointed Junior House Surgeon to the Ingham Infirmary and South Shields and Westoe Dispensary. H. C. Phillips, M.R.C.S., M.&L.S.A., F.G.S., appointed Surgeon to the Westbourne Provident Dispensary. F. E. de Beeho Pim, L.R.C.P., L.R.C.S.Irel., appointed Medical Officer of Health to the Barrowford Urban District Council. M. Ryan, L.R.C.P., L.R.C.S., appointed Medical Officer for the Kilshannig Dispensary District. Mr. A. S. Sorley, appointed Medical Officer of the Toxteth Park Workhouse. H. L. Thurnell, M.A.Camb., M.R.C.S.Eng., L.R.C.P., appointed Medical Officer of Health and Public Analyst for the Borough of Gravesend. O. B. Trumper, M.B., B.Ch.Vict., appointed Medical Officer of Health to the Market Rasen Urban District Council. W. S. Willmore, M.R.C.S.Eng., L.R.C.P.Lond., appointed House Physician to the Queen's Hospital, Birmingham. H. Witham, M.R.C.S., L.R.C.P., appointed Medical Officer for the Kirton District of the Boston Union. S. Woodhams, L.R.C.P.Lond., M.R.C.S.-Eng., appointed Medical Officer to the Gravesend Sanatorium. C. Woodhouse, B.A., M.B., B.C., appointed House Physician to the Paddington Green Children's Hospital.

